# Behavior of a viscoelastic valveless pump: a simple theory with experimental validation

**DOI:** 10.1186/1475-925X-9-42

**Published:** 2010-08-31

**Authors:** Charles F Babbs

**Affiliations:** 1 Weldon School of Biomedical Engineering and Department of Basic Medical Sciences, 1426 Lynn Hall, Purdue University, West Lafayette IN 47907-1246, USA

## Abstract

**Background:**

A valveless pump generates a unidirectional net flow of fluid around a closed loop of soft viscoelastic tubing that is rhythmically compressed at one point. The tubing must have at least two sections with two different stiffnesses. When a short segment of the tube is squeezed asymmetrically at certain frequencies, net flow of fluid around the loop can occur without valves.

**Methods:**

Partial differential equations for the pressures, volumes, and flows define a simple one-dimensional model of such a pump, based upon elementary physical principles. Numerical computations on a personal computer can predict measured net flows.

**Results:**

Net flow varies with the frequency and waveform of compression used to excite the pump, as well as with the site of compression and the stiffness and viscosity of the tubing. Net flows on the order of 1 ml/sec are obtained in a water-filled loop including 46 cm of stiffer plastic (Tygon) laboratory tubing and 70 cm of softer latex rubber tubing.

**Conclusions:**

The heretofore mysterious phenomenon of valveless pumping can be described in terms of classical Newtonian physics, in which viscous damping in the walls of the pump is included. Studying valveless pumps in the laboratory and modeling their behavior numerically provides a low-cost, engaging, and instructive exercise for research and teaching in biomedical engineering.

## Background

Imagine a closed loop of flexible rubber tubing filled with water or a similar incompressible fluid, having nonzero density. In typical laboratory experiments the loop is about 50 to 100 cm in circumference, and the tubing is about 0.5 to 1 cm in diameter. A short segment of the tube is squeezed at a frequency of 1 to 6 Hz. If the wall of the tubing is completely uniform in composition, then a small amount of fluid flows away from the compression point in both directions equally, distending the remainder of the loop slightly. When compression is released, fluid flows back again with no net flow around the loop in either direction. However, if the loop is composed of two different types of tubing with different compliances, one stiffer and one more flexible, then under certain conditions there can be unidirectional net flow around the loop[1-5]. When one end of the softer section of tubing is rhythmically compressed, net flow of fluid around a loop of tubing has been observed in both physical experiments[4,6] and in numerical simulations[1,3-5]. This phenomenon is called *valveless pumping *because forward flow can occur in a closed circulatory system without valves. It is also known as Liebau pumping[7,8].

Valveless pumping is an intriguing phenomenon because the magnitude, and sometimes the direction, of net flow are highly dependent on the frequency of squeezing[1-3]. The frequencies at which valveless pumping has been described fall in the range from 0.2 to 20 Hz [2,3,6,9-11] with maximal net flows occurring near 3 Hz [2], 5 Hz [3,11], 6 Hz [6], or 15 Hz [10], depending on initial conditions. In some cases there is reversal in the direction of net flow with changes in frequency. This behavior recently has been described as "mysterious" by Jung[3] and "difficult to comprehend, even in one dimension" by Manopoulos et al.[10] However, several thinkers have made progress in understanding this curious phenomenon. Auerbach, Moehring, and Maximillian[9] described an analytical solution for the pumping effect in a fluid-filled tube open at both ends with a flow driven by periodically varying the cross sectional area of part of the tube. Recently Hickerson[6,11] has presented a simple one-dimensional model based on wave reflection that can be used to explain the valveless pumping process in a simple and physical way. Other mathematical models of valveless pumping by Jung[1,3] have been based upon Navier-Stokes equations for an incompressible viscous fluid, the elastic boundary equations for the loop of tubing, and the interaction equations for the fluid and elastic boundary. Jung's models predict several interesting phenomena of valveless pumping, including flow reversal with changing frequency.

In general, existing theoretical treatments require complicated systems of equations that do not provide a satisfying intuitive explanation for net unidirectional flow. Further, experimental data validating various mathematical treatments of valveless pumping are extremely scarce. Ottesen[2] provides the most modern and direct comparative data for computer simulations and analogous experiments; however, these involve only one compression frequency and two compression locations.

Accordingly, the present author sought to develop a new mathematical model of valveless pumping, which is as simple as possible, accessible to students, and yet predictive of the results of inexpensive practical experiments within experimental error. The effort revealed an important variable that has been neglected in previous mathematical analyses of valveless pumping[2,3,8-10], namely the viscosity of the tube wall. When the wall sections of the valveless pump are regarded as viscoelastic materials, rather than as simply elastic ones, the fascinating and heretofore mysterious phenomenon of valveless pumping can be understood in terms of one dimensional Newtonian physics.

## Methods

### Governing equations

**Figure 1:**
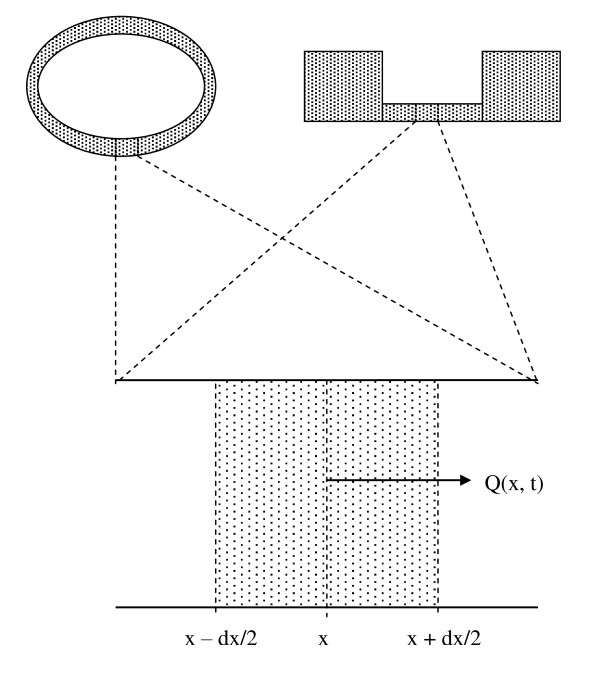
**Segment of a circular or straight valveless pump comprised of a thin walled tube filled with fluid**. A short region of the tube extending from x - dx/2 to x + dx/2 is defined.

Consider a thin-walled viscoelastic tube filled with an incompressible fluid such as water. In one common design of a valveless pump, the tube forms a closed loop. In an alternative design, the tube connects one large reservoir of fluid with another (Figure 1). One section of the tube has a soft wall, and one section of the tube has a stiffer wall. Wall thickness in the soft section is small with respect to the diameter of the tube. The axial distance along the tube is denoted x. The cross sectional area of the tube is denoted A. The flow of fluid along the tube as a function of time, averaged over the cross sectional area, is denoted Q. Part of the soft section is rhythmically squeezed to generate pulsatile flow in the x-dimension. Thus A and Q are functions of both position, x, along the tube and time, t.

Consider a particular point, x, along the length of the tube, as shown in Figure 1. Flow, Q(x, t) is positive in sign when moving to the right. The change in volume of the elastic region extending from x - dx/2 to x+dx/2, over time interval, dt, with varying flow is

(1) $$\frac{\partial \text{A}}{\partial \text{t}}\text{dt}\cdot \text{dx}=\left(\text{Q}(\text{x}-\text{dx})-\text{Q}(\text{x}+\text{dx})\right)\;\;\text{dt}=-\left(\frac{\partial \text{Q}}{\partial \text{x}}\right)\text{​}\;\text{dx}\cdot \text{dt},$$

or simply

$$-\frac{\partial \text{Q}}{\partial \text{x}}=\frac{\partial \text{A}}{\partial \text{t}}.$$

This is the first governing equation for the system.

To derive the second governing equation we apply Newton's second law of motion (force equals mass multiplied by acceleration) to the fluid traveling between x-dx/2 and x+dx/2. Let ρ be constant fluid density. The velocity of the fluid column at point x is Q/A. This is the one dimensional fluid velocity averaged over the cross sectional area. The force F_I _required to overcome the inertia of the fluid is

(2a) $${\text{F}}_{\text{I}}=\rho \;\text{A}\;\text{dx}\cdot \frac{\partial }{\partial \text{t}}\left(\frac{Q}{\text{A}}\right).$$

The force F_R _required to overcome resistance to fluid flow between x-dx/2 and x+dx/2 is given by "Ohm's Law" (force/area equals flow multiplied by resistance). For resistance, R, we have F_R _= AQR. For cylinders with one-dimensional laminar flow we can use Poiseuille's law[12] to estimate the contribution to resistance within a segment of length dx, and internal cross section A, to the laminar flow of fluid having viscosity ν as $\text{R}=\frac{8\pi \nu \;\text{dx}}{{\text{A}}^{2}}\;\;$. Then the force overcoming viscous resistance to fluid flow is

(2b) $${\text{F}}_{\text{R}}=\text{A}\frac{8\pi \nu \;\text{dx}}{{\text{A}}^{2}}\;\;\text{Q},$$

which is relatively small compared to F_I _for typical laboratory scale models with tubing diameter on the order of 1 cm. Hence, Poiseuille's law is sufficiently accurate at this macro scale. (Note also that ν refers to viscosity of the fluid. The viscosity of wall material is described subsequently.)

Now let P represent pressure inside the tube as a function of position and time. The net force on the fluid column between x-dx/2 and x+dx/2 in the positive direction, which over a short distance dx is approximately

$$-\left(\frac{\partial \text{P}}{\partial \text{x}}\right)\;\text{dx}\cdot \text{A}={\text{F}}_{\text{I}}+{\text{F}}_{\text{R}}=\rho \;\text{A}\;\text{dx}\cdot \frac{\partial }{\partial \text{t}}\left(\frac{\text{Q}}{\text{A}}\right)+\text{A}\frac{8\pi \nu \;\text{dx}}{{\text{A}}^{2}}\;\;\text{Q},$$

so that

(2c) $$-\frac{1}{\rho }\frac{\partial \text{P}}{\partial \text{x}}=\frac{\partial }{\partial \text{t}}\left(\frac{\text{Q}}{\text{A}}\right)+\frac{8\pi \nu \;}{\rho \;\text{A}}\;\;\left(\frac{\text{Q}}{\text{A}}\right),$$

or

(2d) $$-\frac{1}{\rho }\frac{\partial \text{P}}{\partial \text{x}}=\frac{\partial \text{u}}{\partial \text{t}}+\frac{8\pi \nu \;}{\rho \;\text{A}}\;\;\text{u}.$$

This is the second governing equation for the system, expressed in terms of the mean fluid velocity u = (Q/A) averaged over the cross section at axial position, x.

Finally, it is necessary to specify the local pressure, P, inside the tube as a function of the pressure difference across the tube wall (P-P_ext_)and the change in local cross sectional area from the resting state (A-A_0_). Since a major discovery of the present research is that the viscosity of the tubing must be accounted, it is instructive to derive the pressure-volume relationship for a soft viscoelastic tube in detail.

First consider a rectangular sheet of wall material subjected to an elongating force in direction y, having initial length y_0_, width L, and thickness h. Let E be Young's modulus of elasticity, which is proportional to the stiffness of the material. Let D be the analogously defined damping modulus (viscosity) of the material. The force required to produce elongation, Δy, of the material is

$$\text{F}=\text{k}\;\Delta \text{y}+\mu \frac{\text{d}\;\Delta y}{\text{dt}},\;\text{with}\;\text{k}=\frac{\text{E}\;\text{h}\;\text{L}}{{\text{y}}_{0}}\;\text{and}\;\mu =\frac{\text{D}\;\text{h}\;\text{L}}{{\text{y}}_{0}}$$

[13,14].

Wall stress, σ, in the viscoelastic sheet is $\sigma =\frac{\text{F}}{\text{h}\;\text{L}}$, and strain $\varepsilon =\frac{\Delta \text{y}}{{\text{y}}_{0}}$. So

$$\sigma =\text{E}\left(\;\varepsilon +\frac{\text{D}}{\text{E}}\frac{\text{d}\varepsilon }{\text{dt}}\right)$$

for the viscoelastic material. When D = 0 in a purely elastic material, σ = Eε.

Consider forming a thin walled tube from this same viscoelastic material with wall thickness h, length L, radius r, and circumference 2πr_0_. Here the "hoop" direction of stretch, as the tube expands, corresponds to y. From the Law of Laplace for thin walled tubes we have wall stress, $\sigma =\frac{\left(\text{P}-{\text{P}}_{\text{ext}}\right)\;\text{r}}{\text{h}}=\text{E}\;\;\left(\;\varepsilon +\frac{\text{D}}{\text{E}}\frac{\text{d}\varepsilon }{\text{dt}}\right)$ in terms of the pressure difference (P-P_ext_) across the tube wall, so

(3a) $$\text{P}={\text{P}}_{\text{ext}}+\frac{\text{Eh}}{\text{r}}\;\left(\;\varepsilon +\frac{\text{D}}{\text{E}}\frac{\text{d}\varepsilon }{\text{dt}}\right).$$

Assuming that the tube wall is made of incompressible material, then the wall volume is constant over time, so hr = h_0_r_0_. Hence, in terms of fundamental material properties and initial conditions, the pressure inside the tube is given by

(3b) $$\text{P}={\text{P}}_{\text{ext}}+\frac{{\text{Eh}}_{0}\;{\text{r}}_{0}}{{\text{r}}^{2}}\;\left(\;\varepsilon +\frac{\text{D}}{\text{E}}\frac{\text{d}\varepsilon }{\text{dt}}\right).$$

For small changes in volume, which are typical for the non-compressed segments in a practical valveless pump, we can relate wall strain to the internal change in volume using V = πLr^2 ^and dV = 2πLr·dr. In turn for small volume changes in a cylindrical tube

$$\varepsilon =\frac{\text{dr}}{{\text{r}}_{0}}=\frac{\text{dV}}{2\;\pi \;{\text{Lr}}_{0}^{2}}=\frac{\text{V}-{\text{V}}_{0}}{2\;\pi \;{\text{Lr}}_{0}^{2}},\;\text{and}\;\frac{\text{d}\varepsilon }{\text{dt}}=\frac{\text{dV}/\text{dt}}{2\;\pi \;{\text{Lr}}_{0}^{2}}.$$

Then, substituting the above for strain, ε, and strain rate, dε/dt, we have for the small signal case

(3c) $$\text{P}={\text{P}}_{\text{ext}}+\frac{{\text{Eh}}_{0}\;}{2\;\pi \;{\text{Lr}}_{0}^{3}}\;\;\left(\;\text{V}-{\text{V}}_{0}+\frac{\text{D}}{\text{E}}\frac{\text{dV}}{\text{dt}}\right).$$

Since the classical resting compliance of an elastic tube [15], ${\text{C}}_{0}=\frac{2\pi \;\text{L}\;{\text{r}}_{0}^{3}}{\text{E}\;{\text{h}}_{0}}$, is the inverse of the lumped constant, we can write a relatively simple expression for a viscoelastic tube,

(3d) $$\text{P}={\text{P}}_{\text{ext}}+\frac{1}{{\text{C}}_{0}}\;\left(\;\text{V}-{\text{V}}_{0}+\frac{\text{D}}{\text{E}}\frac{\text{dV}}{\text{dt}}\right),$$

which differs from the linear P-V relationship for a purely elastic tube only by the term $\frac{\text{D}}{\text{E}}\frac{\text{dV}}{\text{dt}}$. This term, however, turns out to have a significant effect upon the physics of valveless pumping. It also substantially reduces instabilities of numerical solutions for pressure and flow.

External pressure is important to include in (3), because it will be changes in external pressure at the site of compression that drive the valveless pump. The segment that is directly squeezed to drive the pump does have substantial changes in volume, which are not consistent with a small signal assumption. However, in the practical world compression is not radially symmetrical but rather from one side only. In this case buckling of the wall occurs and the deformation of wall areas near the indented region are cantilever-like, and so, roughly linear in mechanical performance. As will be seen, the use of (3) for the compressed segment as well as for the uncompressed segments is sufficient without embellishment for practical purposes. However, the compliance value C_0 _does change abruptly between the stiff and soft sections of tubing in the valveless pump.

The governing equations (1) through (3) of this simple system can be solved numerically to describe the flow and expansion in an elastic tube during valveless pumping. Then calculated results can be compared with measured flows in a working physical model.

### Numerical methods

#### Definitions

**Figure 2:**
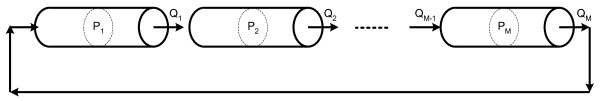
**A theoretical valveless pump, discretized into segments k = 1, 2, ... M, which are connected in a closed loop with segment M joining segment 1**. Here individual segments are shown "exploded", prior to assembly into a loop. Pressures, P, as well as areas, A, are defined at the midpoints of each segment. Flows, Q, are defined as the discharge from the right hand end of each segment.

Let the valveless pump is regarded as a closed loop of fluid-filled tubing, divided into discrete segments numbered 1, 2, 3 ... M. Segment M is connected to segment 1. Let the length of segment k be ΔL_k_. Within each segment wall stiffness and thickness do not change, however, discontinuous boundaries in wall properties may occur between segments at the junction of stiff and soft tubing. Figure 2 illustrates the arrangement of the segments.

In Figure 2 the variable Q_k _indicates the axial flow exiting the right hand end of segment k. Let ${\text{A}}_{\text{k}}^{\text{'}}$ represent the cross sectional area of the fluid slug exiting the right hand end of segment k. Since the segments may have different cross sections, ${\text{A}}_{\text{k}}^{\text{'}}=\min\left({\text{A}}_{\text{k}},{\text{A}}_{\text{k}+1}\right)$ in this discretized system. Also, since the segments may have different lengths, let the length of the fluid slug exiting the right hand end of segment k be $\Delta {\text{L}}_{\text{k}}^{\text{'}}=\frac{1}{2}\left(\Delta {\text{L}}_{\text{k}}+\Delta {\text{L}}_{\text{k}+1}\right)$.

#### Areas and volumes

The area of segment k is obtained from (1) is

(4) $${\text{A}}_{\text{k}}(\text{t})={\text{A}}_{0}+\int_{\;0}^{\;\text{t}}\;\left(\frac{{\text{Q}}_{\text{k}-1}(\text{t})-{\text{Q}}_{\text{k}}(\text{t})\;}{\Delta {\text{L}}_{\text{k}}}\right)\;\text{dt}.$$

In turn,

(5) $${\text{V}}_{\text{k}}(\text{t})={\text{A}}_{\text{k}}(\text{t})\;\Delta {\text{L}}_{\text{k}}.$$

Conservation of volume in the entire closed loop can be checked by computing the aggregate volume ${\text{V}}_{\text{tot}}=\sum_{\text{k}}{\text{A}}_{\text{k}}\;\Delta {\text{L}}_{\text{k}}$ for all segments, k.

#### Instantaneous flows

The values, Q_k _, are obtained by noting that (2) can be re-written as a non-homogenous differential equation with variable coefficients of the form

(6) $$\frac{\text{du}}{\text{dt}}+\text{a}(\text{t})\;\text{u}=\text{b}(\text{t}),$$

where u = Q_k_(t)/A'_k_(t) is the mean fluid velocity exiting the right hand end of segment k at time t, and

$$\text{a}(\text{t})=\frac{8\pi \nu \;}{\rho \;{\text{A}}_{\text{k}}^{\text{'}}(\text{t})}\;,$$

$$\text{b}(\text{t})=\frac{1}{\rho }\frac{{\text{P}}_{\text{k}}-{\text{P}}_{\text{k}+1}}{\Delta {\text{L}}_{\text{k}}^{\text{'}}}\;\text{for}\;\text{k}=1,2,...\text{M}-1,$$

$$\text{and}\;\text{b}(\text{t})=\frac{1}{\rho }\frac{{\text{P}}_{\text{M}}-{\text{P}}_{1}}{\Delta {\text{L}}_{\text{M}}^{\text{'}}}\;\text{for segment M}.$$

Here the relevant cross sectional area for the slug of fluid moving between segment k and segment k+1 is ${\text{A}}_{\text{k}}^{\text{'}}=\min\left({\text{A}}_{\text{k}},{\text{A}}_{\text{k}+1}\right)$, and the relevant length is $\Delta {\text{L}}_{\text{k}}^{\text{'}}=\frac{1}{2}\left(\Delta {\text{L}}_{\text{k}}+\Delta {\text{L}}_{\text{k}+1}\right)$. Flow Q_k _is positive in sign when it leaves the "right hand" end of segment k and enters the "left hand" end of segment k + 1, moving to the right.

As is easily proved by differentiation, the exact general solution of (6) for the initial condition that u = 0 at t = 0 is given by the expression

(7) $$\text{u}=\left({\text{e}}^{-\int_{\;0}^{\;\text{t}}\text{a}(\text{t})\text{dt}}\right)\cdot \left(\int_{\;0}^{\;\text{t}}{\text{e}}^{\int_{\;0}^{\;\text{t}}\text{a}(\text{t})\text{dt}}\cdot \text{b}(\text{t})\;\;\text{dt}\right).$$

Expression (7) is complicated to write algebraically, but easy to compute numerically. In turn, it is a simple matter to find Q_k_(t) = u A'_k_(t). Nonzero flow velocity, u, happens because the pressures in the driven segments are augmented by external pressures. Hence b(t) fluctuates with time. Numerical integration of (4) and (6) is done using the trapezoidal rule: $\int_{\;0}^{\text{t}}\text{f}(\text{t})\;\;\text{dt}≅\sum_{1}^{\text{n}}0.5\;\left(\text{f}(\text{n}\Delta \text{t}-\Delta \text{t})+\text{f}(\text{n}\Delta \text{t})\right)\;\Delta \text{t}$ for the time step of numerical integration, Δt, and t = nΔt.

#### Pressures

To specify pressures for all viscoelastic segments, k, one can use (3d) written in terms of cross sectional area (5), namely

(8) $${\text{P}}_{\text{k}}(\text{t})={\text{P}}_{\text{ext},\text{k}}+\frac{\Delta {\text{L}}_{\text{k}}}{{\text{C}}_{\text{k}}}\;\left({\text{A}}_{\text{k}}(\text{t})-{\text{A}}_{\text{k}}(0)+\frac{\text{D}}{\text{E}}\cdot \frac{{\text{A}}_{\text{k}}(\text{t})-{\text{A}}_{\text{k}}(\text{t}-\Delta \text{t})}{\Delta \text{t}}\right).$$

In (8) P_ext,k _is known because it a defined forcing function. A_k_(0) is known from the initial conditions of the model, and we keep track of area A_k_(t-Δt) at the preceding time step. In this way it is possible to calculate numerically the instantaneous cross sections and flows in all segments of the model at successive time steps Δt for segments k = 1 to M. The required compliance values in (8) are obtained from measured material properties and the initial conditions as

(9) $${\text{C}}_{\text{k}}=\frac{2\pi \;{\text{L}}_{\text{k}}\;{\text{r}}_{0,\text{k}}^{3}}{{\text{E}}_{\text{k}}\;{\text{h}}_{0,\text{k}}}.$$

#### Forcing functions

The model can be driven by applying fluctuating external pressure, P_ext_(t), to one or more compressed segments. In the usual laboratory models this external pressure is always positive. In this case for sinusoidal excitation with angular frequency ω = 2πf t,

(10) $${\text{P}}_{\text{ext}}(\text{t})=\frac{{\text{P}}_{\text{ext-max}}}{2}\;\left(1-\cos(\omega \;\text{t})\right).$$

For convenience in setting the initial conditions one would like to know the exact value of P_ext-max _required to compress the tubing a given amount. That is, starting with P_ext _= 0, the initial resting state, we wish to apply sinusoidal external pressure with a given P_ext-max _until a particular fraction, f_c _, of the resting volume is forced into the rest of the model at maximal compression. A simple scheme for predicting P_ext-max _for practical low frequencies close to 1 Hz is shown in Appendix A. The result in terms of the desired compression fraction and the initial parameters of the system is

(11) $${\text{P}}_{\text{ext-max}}=\frac{2\;{\text{V}}_{1}(0)\;\;{\text{f}}_{\text{c}}}{\frac{{\text{C}}_{1}\;{\text{C}}_{2}}{{\text{C}}_{1}+{\text{C}}_{2}}\;\;\left(\;1+\frac{1}{\sqrt{1+\omega^{2}\;{\text{D}}^{2}/{\text{E}}^{2}}}\right)}$$

where V_1_(0) is the initial resting volume of the compressed segment, C_1 _is the compliance of the compressed segment, calculated using (9), and C_2 _is the lumped compliance of all uncompressed segments.

#### Numerical integration

In this way one can model a viscoelastic valveless pump driven by time varying external pressure. The numerical computation, after specifying initial conditions, consists of computing at successive time steps the areas for each segment using (4), the flows exiting each segment as the product of flow velocity (7) and area A'_k_(t), the internal pressures of each segment using (8), and the external pressure on each segment using (10). Known variations in external pressure drive the pump. The resulting numerical integrations give instantaneous values for area, flow, and pressure of each segment, k, as time, t progresses. In the results that follow mean flow was computed as average flow in all segments between the 10^th ^and 20^th ^compressions. The calculations were done using Microsoft Visual Basic Macros within a Microsoft Excel spreadsheet to perform numerical integration. For convenience and accuracy, all variables were converted to units of grams, centimeters, and seconds.

#### Initial conditions

Input parameters for the numerical model matched those of the physical model. For the results reported here, the value Eh, the product of Young's modulus and wall thickness for the soft section was 2.2 × 10^5 ^dynes/cm^2^. The value of D/E was 0.0083 sec. These values are means of five sets of measurements on latex rubber tubing, as subsequently described under experimental methods. The value of Eh for the stiff section was taken as 30 times that of the soft section (i.e. the stiff section is essentially rigid). Results are essentially similar when Eh for the stiffer section is more than 10 fold greater than Eh for the softer section.

To model the particular physical pump described herein, the soft section of tubing was represented by 41 discrete segments, each 1.7 cm long (70 cm total). The stiff section of tubing was represented by one segment 46 cm long. The lengths of segments in the numerical model were chosen to be much less than the impulse wavelength at the highest frequency tested, where impulse wavelength is computed as the product of cycle time (1/frequency) and pulse wave speed calculated from the Moens-Korteweg equation[16-18] for the wave speed, s, along the tube with radius, r, namely, $\text{s}=\sqrt{\text{Eh}/(2\;\rho \;\text{r})}$. The time step for numerical integration was less than 0.1 msec and sufficiently small to permit stability of solutions that do not differ substantially when time step is halved. The model was driven by external positive pressure (10) applied to the 7^th ^segment (10 cm) from the stiff-soft junction. The width of this compression zone (1.7 cm) and the compression fraction (0.95) used to compute theoretical results were similar to those used in corresponding physical experiments.

#### Numerical accuracy

The accuracy of numerical solutions was verified by computing conservation of volume routinely, by comparing pulse wave velocity with the analytical result of the Moens-Korteweg equation, and by computing conservation of energy for reflected pulse waves for no-leak test cases with an extremely small tube radius in part of the loop, to represent a closed, straight tube. Comparison of simple Euler forward difference solutions (not described here) with exact solutions (4) and (7) provided a further test of numerical accuracy, and comparison of theoretical and experimental results provided a final test of the validity of the theory and associated simplifying assumptions.

### Experiment

#### Construction of a working valveless pump

**Figure 3:**
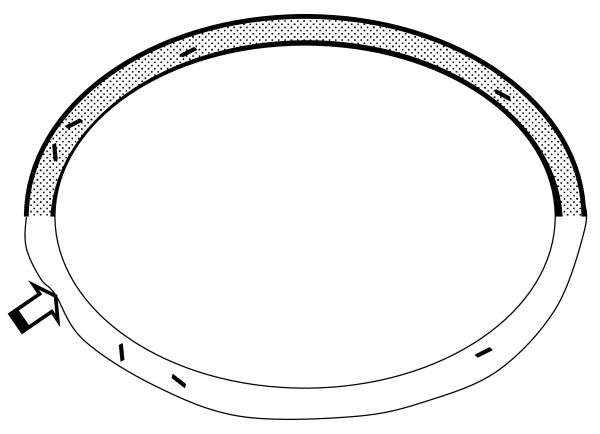
**Water-filled experimental valveless pump constructed of latex rubber and plastic laboratory tubing**. The stiff section (top) is 46 cm long. The soft section (bottom) is 70 cm long. A "T" connector at the midpoint of the top section (not shown) and stopcock allowed for filling and clearance of air bubbles. Suspended bits of colored paper provided markers for tracking average fluid velocity. Compression point is indicated by broad arrow.

A practical valveless pump was constructed using 1/4 inch amber latex tubing for the soft section (Penrose surgical drain) and 1/4 inch O.D. colorless Tygon laboratory tubing for the stiff section (Figure 3). The wall thickness of the latex tubing was approximately 0.04 cm and the wall thickness of the Tygon tubing was approximately 0.2 cm. The inner diameter of the inflated soft section was 0.6 cm and the inner diameter of the stiff section was 0.4 cm. A plastic T fitting having similar internal diameter and connected to a stopcock was placed at the midpoint of the stiff section to provide for filling and drainage of fluid using a syringe. The stopcock also allowed for evacuation of bubbles. The Penrose drain overlapped the ends of the Tygon tubing and was secured with thread to prevent leaks. The length of the stiff section was 46 cm and the length of the soft section was 70 cm. The circuit was filled with tap water, in which were suspended small pieces of colored paper towel to serve as visible markers of fluid flow.

#### External compression

The valveless pump was driven by finger compression of the soft section against a hard, horizontal surface. Refill of the compressed segment was passive. The width of the compression zone was 1.7 cm. Sufficient finger pressure was applied to almost completely occlude the latex tubing at the instant of maximum compression. Two compression waveforms could be easily implemented. One was a sinusoidal waveform, created by smoothly moving the compressing finger up and down without losing contact with the tubing. The other was a rectangular waveform, created by holding the finger one diameter above the tubing and performing sharp compressions with equal up and down times and a quick release. Spontaneous recoil of the tubing was sufficient to generate an approximately rectangular waveform of compression with a 50% duty cycle. The compression zone extended from 10 to 11.7 cm away from either end of the stiff section. Alternate ends of the soft section were compressed on alternate trials.

#### Measurement of average flow and frequency

Mean flow could be determined from the transit time of a fluid marker passing through the clear Tygon section of the valveless pump. Mean flow is calculated as the product of cross sectional area and distance moved through the stiff section, divided by transit time. Small pieces of colored paper towel provided long-lasting visible markers that did not cling to the sides of the tubing or obstruct flow. Their motion can be observed and measured using the naked eye. Compression frequency is determined as the number of compressions divided by transit time. Time is measured using a stopwatch.

#### Material properties of the soft section

Young's modulus of stiffness, E, and the damping modulus, D, were determined from independent tests on the Penrose drain material used for the soft section. These parameters are needed for the theoretical calculations of pressures in (3) and (8). Since the thickness of this thin walled tubing is difficult to measure accurately, the lumped product of E and wall thickness (Eh) can be determined by hanging a known weight of mass, m, in the range of 100 to 300 grams from a 10 cm vertical length, L_0_, of Penrose drain and observing the equilibrium extension, Δx*, under 1 G. Here we regard the tubing as a rectangular elastic solid having a total width, w, equal to twice the flattened diameter. Young's modulus, E, is stress/strain or

(12) $$\text{E}=\frac{\text{m}\;\text{g}}{\text{h}\;\text{w}}\cdot \frac{1}{\left(\Delta \text{x}*/{\text{L}}_{0}\right)}\;\text{or}\;\text{Eh}=\frac{\text{m}\;\text{g}\;{\text{L}}_{0}}{\text{w}\;\Delta \text{x}*}.$$

The value of Eh for the theoretical curves plotted in the present paper was taken as the mean of 5 measurements using (12) on Penrose drain latex tubing. This value (± SD) was 221,000 ± 31000 dynes/cm^2^.

The damping modulus, D, or in particular the ratio D/E, can be determined from observations of the decay in amplitude of oscillations when the mass tethered by the tubing under study is released from the un-stretched level x = 0 with the upper end of the tubing solidly secured to the edge of a table. When care is taken to prevent swinging, the mass undergoes up and down damped sinusoidal oscillation in one dimension according to the governing equation

$$\begin{array}{l} \text{m}\ddot{\text{x}}=\text{mg}-\frac{\text{Eh}\;\text{w}}{{\text{L}}_{0}}\text{x}-\frac{\text{Dh}\;\text{w}}{{\text{L}}_{0}}\dot{\text{x}}\;\text{or}\\ \ddot{\text{x}}=\text{g}-\frac{\text{Eh}\;\text{w}}{{\text{L}}_{0}\text{m}}\text{x}-\frac{\text{Dh}\;\text{w}}{{\text{L}}_{0}\text{m}}\dot{\text{x}}=\text{g}-\frac{\text{Eh}\;\text{w}}{{\text{L}}_{0}\text{m}}\left(\text{x}-\frac{\text{D}}{\text{E}}\;\dot{\text{x}}\right), \end{array}$$

where $\dot{\text{x}}$ represents the first time derivative and $\ddot{\text{x}}$ represents the second time derivative. Substituting expression (12), we can solve for the motion of the mass numerically in small time steps Δt, using

(13a) $$\ddot{\text{x}}=\text{g}-\frac{\text{g}}{\Delta \text{x}*}\left(\text{x}-\frac{\text{D}}{\text{E}}\;\dot{\text{x}}\right)$$

(13b) $$\dot{\text{x}}=\dot{\text{x}}+\ddot{\text{x}}\;\Delta \text{t},\;\text{and}$$

(13c) $$\text{x}=\text{x}+\dot{\text{x}}\;\Delta \text{t}$$

with initial conditions at time zero $\text{x}=\dot{\text{x}}=\ddot{\text{x}}=0$. Here g is a known constant, and Δx* is measured after damped oscillations subside. Hence there is only one free parameter in (13), namely D/E.

To estimate the damping ratio D/E one can solve (13 a - c) in a simple spreadsheet program for different values of D/E and observe the plotted values of displacement, Δx, as a function of time. When D/E is zero there is no damping and the motion is perfectly sinusoidal. When D/E is large the motion is highly damped. By trial-and-error one can quickly obtain a good estimate for D/E, for which the calculated number of oscillations matches the observed value. Since the observed number of oscillations is quite sensitive to changes in damping, this process gives the value of D/E for the system to within about 10 percent. The value of D/E for the theoretical curves plotted in the present paper was taken as the mean of 5 such measurements on Penrose drain latex tubing. This value (± SD) was 0.0083 ± 0.0012 sec.

Once values for the stiffness-thickness product, Eh, and the damping to elastic modulus ratio, D/E, are obtained experimentally for the wall material of the soft section, the one-dimensional model embodied in equations (1), (2), and (3) can be solved with no free parameters.

## Results and discussion

**Figure 4:**
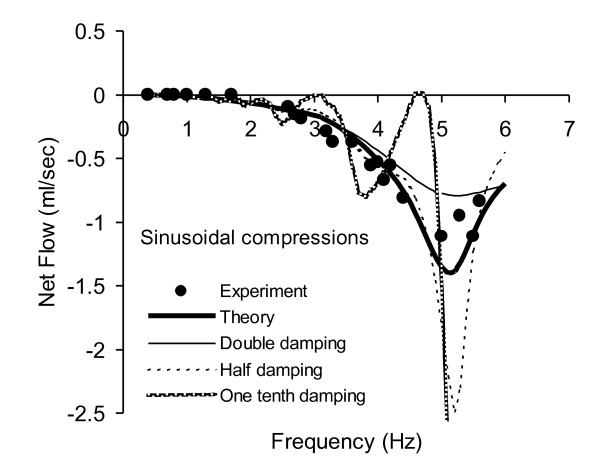
**Calculated vs. measured mean flow for smooth, sinusoidal compression of the practical valveless pump in Figure 3**. Solid curve represents a 42 compartment numerical model incorporating measured material properties and dimensions. Discrete points are individual measurements of flow, not averages. Reduction in damping modulus by a factor of one half produces large differences in predicted flows near 5 Hz.

Mean flow for a 42 compartment numerical model, using measured elastic and damping moduli and the dimensions of the physical model, are shown a heavy smooth curve in Figure 4. Measured values of mean flow for sinusoidal finger compressions are plotted as discrete points. Each data point represents a single observation, not an average, illustrating the stability of the phenomenon. The positive direction of flow is taken as that from the compression point to the center of the soft section. In these experiments flow nearly always occurred away from the center of the soft section, and so plotted values are negative. There is good agreement between theory and experiment when the measured value of the damping ratio D/E = 0.0083 is used in the theoretical calculations. The thinner smooth curves in Figure 4 illustrate theoretical values of mean flow with double, one-half, or one-tenth the measured value of D/E. (Computations with zero wall viscosity tended to produce unstable results.) It is clear that correct prediction of mean flow at frequencies near 5 Hz is highly dependent upon wall viscosity. When viscosity is halved, net flow doubles near 5 Hz. When viscosity is one-tenth normal, theory diverges greatly from experiment. In this case maximal net flow (off scale in Figure 4) is five times experimentally measured flow at 5.3 Hz.

**Figure 5:**
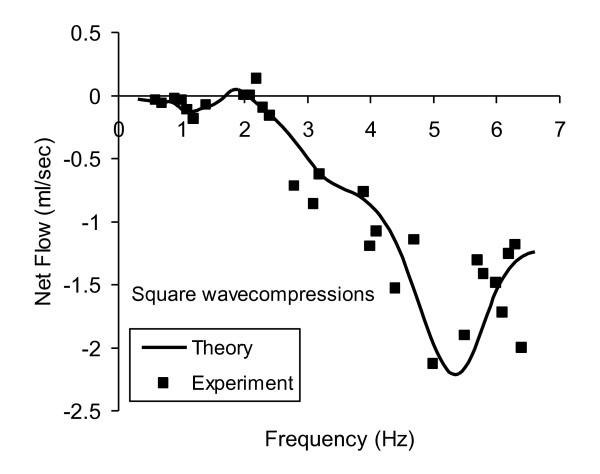
**Calculated vs. measured mean flow for quick, sharp rectangular wave compressions**. Other details similar to Figure 4.

Figure 5 compares results for theory and experiment using more abrupt rectangular waves of external compression as a function of time. In this case a rectangular waveform was substituted for the cosine wave in expression (10) for theoretical calculation of external pressure. A sharper compression style produces larger flows both theoretically and experimentally. Subtle differences in the flow versus frequency spectrum include significant negative flow near 1 Hz, measurable positive flow near 2 Hz, and a shoulder in the spectrum near 4 Hz. One-dimensional viscoelastic theory using the correct value of wall viscosity can predict subtle changes in the shape of the mean flow versus frequency spectrum with use of different compression waveforms. These results, obtained with simple manual compression of the soft segment, could be improved and extended using a motor driven piston for compression.

The goal of this investigation was to develop the simplest possible mathematical treatment of valveless pumping in one dimension that would predict experimental results. Inclusion of the wall viscosity term led to good agreement of theoretical and experimental values for net flow. There was no need to consider fluid velocity profiles in the radial dimension of the tube. There was no need to consider nonlinear elastic properties of the tube walls. The frequencies of compression of the soft segment predicted to produce maximal one-way flow are similar to those reported previously[2,3,6,10,11].

The present mathematical treatment provides a more compact explanation of the phenomenon of valveless pumping, heretofore described as a mysterious and hard to comprehend[3,10]. The underlying physics can be represented by three simple equations that describe volume conservation, Newton's second law of mass and acceleration, and a pressure volume function for a viscoelastic tube. To understand valveless pumping properly, however, it is important to regard the pump as viscoelastic and not simply elastic. Wall viscosity in fact can dominate the pressure-volume function in practical, real-world latex tubes. Other theoretical treatments of valveless pumping have included the viscosity of the water inside the pump, but not the viscosity of the tubing in the wall of the pump, which is on the order of 10^6 ^times greater than that of water (10^4 ^dyne sec/cm^2 ^vs 0.01 dyne sec/cm^2^). Previous theoretical models retain small, higher order terms from the calculation of force = d(mv)/dt. However, these terms are not important in predicting the results of practical experiments[2,9,14], whereas wall viscosity is quite important.

## Conclusions

Valveless pumping is an intriguing phenomenon that has attracted a number of thinkers in the past. It appears at first to be almost magical and has remained difficult to comprehend[10]. A missing variable in previous analyses is the viscosity of the wall of the pump. Inclusion of realistic damping from wall viscosity dispels some of the mystery of valveless pumping and allows for better predictions of how such pumps might operate in practice. The simple one-dimensional physics and experimental methods described here make it easy and inexpensive for students and teachers of biomedical engineering to explore this interesting phenomenon. The effect is easy to demonstrate experimentally and would make a good classroom demonstration.

## Competing interests

The author declares he has no competing interests.

## Authors' contributions

CB is the only author and is responsible for all aspects of the research and the intellectual and technical content of the manuscript.

## Appendix A: maximal external sinusoidal pressure as a function of the desired compression fraction

Consider the volume of all directly compressed segments at time zero as V_1_(0) and the generally larger volume of the remainder of the model at time zero as V_2_(0). The lumped compliance of V_1 _is C_1 _, which is connected to the lumped compliance of the rest of the model, C_2_. As compression of V_1 _occurs fluid is forced into V_2_. Expansion happens in the radial dimension orthogonal to the long axis. Inertia and momentum changes in this dimension are small; hence we need to consider viscoelastic forces only. From (3d) we have

(A1) $${\text{P}}_{1}={\text{P}}_{\text{ext}}+\frac{1}{{\text{C}}_{1}}\;\left(\;{\text{V}}_{1}-{\text{V}}_{1}(0)+\frac{\text{D}}{\text{E}}\frac{{\text{dV}}_{1}}{\text{dt}}\right)\;\text{and}\;{\text{P}}_{2}=\frac{1}{{\text{C}}_{2}}\;\left(\;{\text{V}}_{2}-{\text{V}}_{2}(0)+\frac{\text{D}}{\text{E}}\frac{{\text{dV}}_{2}}{\text{dt}}\right).$$

There is zero external pressure on section 2. For conservation of volume V_2_(t)-V_2_(0) = -(v_1_(t)-V_1_(0)), and $\frac{{\text{dV}}_{2}}{\text{dt}}=-\frac{{\text{dV}}_{\text{1}}}{\text{dt}}$. Also we assume P_1_(t) = P_2_(t). This is essentially a low frequency assumption, which is reasonable for typical macroscopic laboratory models. Then

$$-\frac{1}{{\text{C}}_{2}}\;\left({\text{V}}_{1}-{\text{V}}_{1}(0)+\frac{\text{D}}{\text{E}}\frac{{\text{dV}}_{\text{1}}}{\text{dt}}\right)={\text{P}}_{\text{ext}}+\frac{1}{{\text{C}}_{1}}\;\left({\text{V}}_{1}-{\text{V}}_{1}(0)+\frac{\text{D}}{\text{E}}\frac{{\text{dV}}_{\text{1}}}{\text{dt}}\right)$$

and for positive external sinusoidal pressure on section 1 we have,

(A2) $$-\left(\frac{1}{{\text{C}}_{1}}+\frac{1}{{\text{C}}_{2}}\right)\;\left(\;{\text{V}}_{1}-{\text{V}}_{1}(0)+\frac{\text{D}}{\text{E}}\frac{{\text{dV}}_{\text{1}}}{\text{dt}}\right)=\frac{{\text{P}}_{\text{ext-max}}}{2}\;\left(\;1-\cos(\omega \;\text{t})\right).$$

The solution for this first order ordinary differential equation in V_1 _is

(A3) $${\text{V}}_{1}(\text{t})={\text{V}}_{1}(0)-\frac{{\text{P}}_{\text{ext-max}}}{2}\cdot \frac{{\text{C}}_{1}\;{\text{C}}_{2}}{{\text{C}}_{1}+{\text{C}}_{2}}\;\;\left(\;1-\frac{\cos(\omega \;t-\varphi )}{\sqrt{1+\omega^{2}\;{\text{D}}^{2}/{\text{E}}^{2}}}-\frac{\omega^{2}\;{\text{D}}^{2}/{\text{E}}^{2}}{1+\omega^{2}\;{\text{D}}^{2}/{\text{E}}^{2}}\;\;{\text{e}}^{-\frac{\text{E}}{\text{D}}\;\text{t}}\right),$$

where $\varphi =\tan^{-1}\left(\frac{\omega \;D}{E}\right)$.

After the rapidly diminishing exponential term has vanished, the minimum volume, corresponding to maximal external compression at low frequencies occurs at time points when ωt-ϕ = π or

(A4) $${\text{V}}_{\text{min}}={\text{V}}_{1}(0)-\frac{{\text{P}}_{\text{ext-max}}}{2}\cdot \frac{{\text{C}}_{\text{1}}\;{\text{C}}_{\text{2}}}{{\text{C}}_{\text{1}}{\text{+C}}_{\text{2}}}\;\;\left(\;1+\frac{1}{\sqrt{1+\omega^{2}\;{\text{D}}^{2}/{\text{E}}^{2}}}\right).$$

For compression fraction ${\text{f}}_{\text{c}}=1-\frac{{\text{V}}_{\text{min}}}{{\text{V}}_{1}(0)}$, we have

(A5) $${\text{f}}_{\text{c}}=\frac{{\text{P}}_{\text{ext-max}}}{2{\text{V}}_{1}(0)}\cdot \frac{{\text{C}}_{1}\;{\text{C}}_{2}}{{\text{C}}_{1}+{\text{C}}_{2}}\;\;\left(\;1+\frac{1}{\sqrt{1+\omega^{2}\;{\text{D}}^{2}/{\text{E}}^{2}}}\right),$$

and we can specify

(A6) $${\text{P}}_{\text{ext-max}}=\frac{2{\text{V}}_{1}(0)\;\;{\text{f}}_{\text{c}}}{\frac{{\text{C}}_{\text{1}}\;{\text{C}}_{\text{2}}}{{\text{C}}_{\text{1}}{\text{+C}}_{\text{2}}}\;\;\left(\;1+\frac{1}{\sqrt{1+\omega^{2}\;{\text{D}}^{2}/{\text{E}}^{2}}}\right)}$$

in terms of the desired compression fraction and the initial parameters of the system. In this way we can model a viscoelastic valveless pump driven by time varying external pressure that produces a specified compression fraction.

## Appendix B: problems and exercises for students

1. Are there sweet spots and dead spots for compression along the soft section? Explore both numerically and experimentally net flows as a function of the site of finger compression along the soft section. Flow should be zero when compression is applied at the exact midpoint--an obvious dead spot. Are there circumstances in which there are other dead spots and intervening sweet spots, which produce flow maxima? In numerical simulations try exploring substantially longer soft sections and substantially softer materials in these sections. In such cases the pulse transit time across the soft section is much longer than that in the original physical model described above. Consider the analogy of a standing wave. Experimentally, what happens if you substitute ice water for room temperature water in the valveless pump and keep the apparatus as cool as possible? Can you explain the findings on the basis of increased viscosity of the soft rubber tubing?

2. Suppose that valveless pumping has something to do with standing wave patterns in the soft section, in which the driving frequency of compression is the inverse the round-trip transit time for a pulse wave traversing the section and reflected at the stiff-soft boundaries. Explore cases in which Young's modulus of the stiff section is at least 10 times greater than that of the soft section, so that there is essentially complete reflection of the pulse waves. Use the Moens-Korteweg equation to estimate pulse wave speed, s, in the soft section, namely, $\text{s}=\sqrt{\text{Eh}/(2\;\rho {\text{r}}_{0})}$. What happens in a valveless pump with a soft section of length L, excited at frequency, f, when 2L = s/f, that is, when the period of excitation is equal to the round-trip transit time? What happens when the period of excitation is twice or half the round trip-transit time? Can you predict peaks in the net flow vs. frequency spectrum? Is the pattern of sweet spots and dead spots in problem 1 related to the "impulse wavelength" (s/f), which is the wave speed multiplied by the wave period?

## References

[B1] Jung E Simulations of valveless pumping using the immersed boundary method PhD 1999 New York University, Courant Institute of Mathematical Sciences

[B2] Ottesen JT Valveless pumping in a fluid-filled closed elastic tube-system: one-dimensional theory with experimental validation J Math Biol 2003 46 309 332 10.1007/s00285-002-0179-1 12673509

[B3] Jung E Peskin CS Two-dimensional simulations of valveless pumping using the immersed boundary method SIAM J Sci Comput 2001 23 19 45 10.1137/S1064827500366094

[B4] Moser M Huang J Schwartz G Kenner T Noordergraaf A Impedance defined flow, generalisation of William Harvey's concept of the circulation--370 years later International journal of cardiovascular medicine and science 1998 1 205 211

[B5] Thomann H A simple pumping mechanism in a valveless tube Journal of Applied Mathematics and Physics (ZAMP) 1978 29 169 177 10.1007/BF01601511

[B6] Hickerson AI Rinderknecht D Gharib M Experimental study of the behavior of a valveless pump Experiments in Fluids 2005 38 534 540 10.1007/s00348-005-0946-z

[B7] Kenner T Moser M Tanev I Ono K The Liebau-effect or on the optimal use of energy for the circulation of blood Scripta Medica (BRNO) 2000 73 9 14

[B8] Borzi A Propst G Numerical investigation of the Liebau phenomenon Zeitschrift fur angewandte Mathematik und Physik (ZAMP) 2003 54 1050 1072 10.1007/s00033-003-1108-x

[B9] Auerbach D Moehring W Moser M An Analytic Approach to the Liebau Problem of Valveless Pumping Cardiovascular Engineering 2004 4 201 207 10.1023/B:CARE.0000031549.13354.5e

[B10] Manopoulos CG Mathioulakis DS Tsangaris SG One-dimensional model of valveless pumping in a closed loop and a numerical solution Physics of fluids 2006 18 1 16 10.1063/1.2165780

[B11] Hickerson AI Gharib M On the resonance of a pliant tube as a mechanism of valveless pumping Journal of Fluid Mechanics 2006 555 141 148 10.1017/S0022112006009220

[B12] Schmid-Schonbein H Greger R Hemorheology Comprehensive Human Physiology 1996 2 Berlin, Heidelberg: Springer-Verlag 1747 1792

[B13] Fung YC Biomechanics: mechanical properties of living tissues 1981 New York: Springer-Verlag

[B14] Fung YC Biomechanics: Circulation 1997 2 New York: Springer-Verlag

[B15] Posey J Geddes L Measurement of the modulus of elasticity of the arterial wall Cardiovascular Research Center Bulletin 1973 11 83 103

[B16] Brennan EG O'Hare NJ Walsh MJ Transventricular pressure-velocity wave propagation in diastole: adherence to the Moens-Korteweg equation Physiol Meas 1998 19 117 123 10.1088/0967-3334/19/1/011 9522393

[B17] Callaghan FJ Babbs CF Bourland JD Geddes LA The relationship between arterial pulse-wave velocity and pulse frequency at different pressures J Med Eng Technol 1984 8 15 18 10.3109/03091908409032067 6716443

[B18] Korteweg DJ Uber die Fortpflanzungsgeschwindigkeit des Schalles in elastischen Rohren Ann Phys Chem 1878 5 525 542 10.1002/andp.18782411206

